# Addressing the Know-Do Gap in Adolescent HIV: Framing and Measuring Implementation Determinants, Outcomes, and Strategies in the AHISA Network

**DOI:** 10.1007/s10461-023-04021-3

**Published:** 2023-03-11

**Authors:** Kristin Beima-Sofie, Irene Njuguna, Tessa Concepcion, Stephanie M. DeLong, Geri Donenberg, Brian C. Zanoni, Dorothy Dow, Paula Braitstein, Anjuli Wagner

**Affiliations:** 1 Department of Global Health, University of Washington, Seattle, WA USA; 2 Department of Epidemiology, Johns Hopkins Bloomberg School of Public Health, Baltimore, MD USA; 3 Medical Research Department, Kenyatta National Hospital, Nairobi, Kenya; 4 Center for Dissemination and Implementation Science (CDIS), Department of Medicine, University of Illinois at Chicago, Chicago, IL USA; 5 Departments of Medicine and Pediatric Infectious Diseases, Emory University School of Medicine, Atlanta, GA USA; 6 Children’s Healthcare of Atlanta, Atlanta, GA USA; 7 Department of Pediatrics, Infectious Diseases, Duke University School of Medicine, Durham, NC USA; 8 Duke Global Health Institute, Durham, NC USA; 9 Division of Epidemiology, Dalla Lana School of Public Health, University of Toronto, Toronto, Canada; 10 Division of Epidemiology and Medical Statistics, School of Public Health, College of Health Sciences, Moi University, Eldoret, Kenya

**Keywords:** Implementation science, Know-do gap, Adolescent/youth HIV

## Abstract

Implementation science (IS) uses systematic methods to close gaps between research and practice by identifying and addressing barriers to implementation of evidence-based interventions (EBIs). To reach UNAIDS HIV targets, IS can support programs to reach vulnerable populations and achieve sustainability. We studied the application of IS methods in 36 study protocols that were part of the Adolescent HIV Prevention and Treatment Implementation Science Alliance (AHISA). Protocols focused on youth, caregivers, or healthcare workers in high HIV-burden African countries and evaluated medication, clinical and behavioral/social EBIs. All studies measured clinical outcomes and implementation science outcomes; most focused on early implementation outcomes of acceptability (81%), reach (47%), and feasibility (44%). Only 53% used an implementation science framework/theory. Most studies (72%) evaluated implementation strategies. Some developed and tested strategies, while others adapted an EBI/strategy. Harmonizing IS approaches allows cross study learning and optimization of delivery of EBIs and could support attainment of HIV goals.

**Supplementary Information:**

The online version contains supplementary material available at 10.1007/s10461-023-04021-3.

## Introduction

Adolescents and young adults (AYA) remain a key priority population for the achievement of global HIV targets. Research over the past decade has highlighted significantly poorer clinical outcomes across HIV testing, linkage to care, initiation of treatment and viral suppression among AYA compared to adult populations [1–3]. In addition, HIV incidence among AYA remains high, especially among adolescent girls and young women (AGYW), with an estimated 5000 new infections occurring among AGYW each week [4]. Although interventions for improving poor HIV outcomes among AYA exist, the majority have yet to be scaled up and implemented programmatically. To reach global HIV targets for AYA, it is critical to identify and address unique gaps in the translation and scale-up of evidence-based interventions (EBIs) among this key population. Critical gap areas for this population include adherence and retention, transitional care from pediatric to adult services, integration of mental health and sexual and reproductive health services into HIV services, and prevention of new infections [5].

Implementation science (IS) uses systematic methods to close the know-do gap that exists between research and clinical practice by identifying and addressing barriers to the implementation of EBIs. To accelerate progress towards UNAIDS 95-95-95 goals, global focus has shifted to IS to reach the most vulnerable populations, as well as sustain changes made to optimize HIV clinical outcomes [6, 7]. IS methods can address critical gaps, particularly for children and adolescents, in whom evidence is largely lacking and predominantly extrapolated from adult studies [8]. While this approach has enabled faster implementation of EBIs for this marginalized population, it may result in less effective implementation if there is inflexibility to adapt to the specific unique needs of the population which may result in lack of effectiveness [8]. By identifying the processes used in implementation, and measuring contextual factors influencing implementation, IS provides insight into the heterogeneity observed in implementation of EBIs across varied settings and helps identify how to optimize and adapt EBIs for maximum impact.

The emergent field of IS has wide variation in how measures are defined, applied and studied [9]. Frameworks provide a way to harmonize the use of IS measures and compare IS outcomes across a wide range of settings and populations. Using consistent approaches to measure and evaluate implementation processes and contextual influences on implementation of EBIs could be especially valuable for AYA, where rapid translation of research to clinical practice has the potential to significantly improve health for a future generation. In addition, IS data collection tools have largely been qualitative, with only a few quantitative tools validated in resource limited settings [10, 11]. Given the global distribution of the epidemic, understanding how IS concepts are applied in AYA HIV research, as well as how IS measures, outcomes and determinants are adapted for LMIC settings, is a key strategy to understanding how to end the HIV epidemic. Harmonizing IS measures across studies and settings, developing reliable and valid ways of assessing IS measures, and identifying when and how specific measures are selected, is critical to support innovations in the field of IS, and areas of focus for future AYA research. In this paper, we review ongoing AYA implementation research in the Adolescent HIV Prevention and Treatment Implementation Science Alliance (AHISA) network to identify IS measures, frameworks and outcomes used across the network and determine gaps in methodology and rigor.

## Methods

### Study Context

In 2017, to catalyze IS research within the field of adolescent HIV, the NIH convened the Adolescent HIV Prevention and Treatment Implementation Science Alliance (AHISA), a collaboration where researchers, program implementers, and policymakers could share experiences and exchange ideas to facilitate effective implementation of EBIs in the sub-Saharan context [12]. Principal and co-investigators of funded projects (study teams) were eligible to apply for AHISA membership if their research included evaluation of one or more domains within the HIV care continuum and focused on AYA in Africa. AHISA is currently composed of 26 study teams, conducting one or more research studies in 11 countries in Africa, including 5 countries with the highest prevalence of adolescent HIV globally (South Africa, Nigeria, Kenya, Uganda, Tanzania) [13].

### Study Design & Data Collection

This review aimed to summarize ongoing studies conducted by AHISA members and characterize implementation and clinical outcomes measured, EBIs and implementation strategies tested, and identify gaps in the scientific agenda of IS for AYA across the HIV prevention and HIV care cascades. We presented the review’s aim and purpose to all AHISA member study teams during the 5th Annual AHISA Meeting (February 11–12, 2021). We requested study protocols and protocol manuscripts via email from the PI’s of all 26 study teams. Each AHISA study team provided between 1 and 3 study protocols for review.

### Analysis

ATLAS.ti version 9 (Scientific Software Development GmbH) supported coding and analysis of submitted study documents. Codes were developed by the authors to extract information related to study context (study design, population, geographical setting), EBIs and clinical or efficacy/effectiveness outcomes assessed, implementation strategies tested, and implementation outcomes and/or determinants measured. We utilized Proctor’s Implementation Outcome Framework (IOM) [14] and the Reach, Effectiveness, Adoption, Implementation, and Maintenance (RE-AIM) framework [15] to define and classify outcomes (Table 1). IS outcomes were first identified if explicitly named in study documents. These outcomes were reviewed by manuscript co-authors for consistent interpretation between studies and re-categorized as needed to match definitions in Table 1. Additional IS outcomes described in study documents, but not explicitly named, were also categorized by co-authors using IOM and RE-AIM definitions in Table 1. All study populations that included any age bands between 10 and 24 years of age were grouped as AGYW if defined as female gender only, youth with HIV (YLH) if living with HIV, or youth if they included both populations living with and without HIV. Those that included adolescents (ages 10–19) only were classified as adolescents living with HIV (ALH). Where possible, we mapped implementation strategies to the Expert Recommendations for Implementing Change (ERIC) [16].

**Table 1:** Implementation science outcome definitions and examples from AHISA network studies

Implementation outcomes	Proctor or RE-AIM	Proctor/RE-AIM definition*	Level of operationalization	Example of application to adolescent HIV from AHISA network studies
Acceptability	Proctor	“…the perception among implementation stakeholders that a given treatment, service, practice, or innovation is agreeable, palatable, or satisfactory.”	Individual consumer Individual provider	“Small focus group discussions (FGDs) will be conducted with a subset of study participants in the intervention arm at month 6 to discuss the acceptability of the intervention and its effects on relationship dynamics and the decision to use PrEP.” Study: *Tu’Washindi* [17]
Adoption	RE-AIM, Proctor	RE-AIM: “Proportion of settings, practices, and plans that will adopt the intervention” Proctor: “…the intention, initial decision, or action to try or employ an innovation or evidence-based practice. Adoption also may be referred to as ‘uptake.’”	Individual provider (Proctor) Organization or setting (Proctor, RE-AIM)	“This resulted in 57 successfully trained peer—navigators being employed for 24 h work per week to co-create and implement the intervention in their areas.” Study: *Thetha Nami* [18]
Appropriateness	Proctor	“…the perceived fit, relevance, or compatibility of the innovation or evidence-based practice for a given practice setting, provider, or consumer; and/or perceived fit of the innovation to address a particular issue or problem.”	Individual consumer Individual provider Organization or setting	“Although originally designed and tested with African-American 14–18 year-old girls in the United States, our systematic approach to adaptation for the South African local context increases confidence in its local relevance. This study is among the first to evaluate the impact of a female caregiver—adolescent girl and young woman intervention on HIV testing and counseling and PrEP uptake using a rigorous and culturally adapted trial of sufficient size to detect effects on adolescent girl and young woman sexually transmitted infection incidence and explore HIV incidence.” Study: *SA Imara* [19]
Costs	Proctor	“…the cost impact of an implementation effort.”	Provider or providing institution	“Where the data from Aim 3 indicate a positive effect of interventions, the modelling framework will be used to undertake a formal cost-effectiveness analysis. This analysis will use the actual costs and observed outcomes and will project the stream of benefits that could accrue to the young women and their partners and the indirect population-level benefits. Summary measures of benefit will include infections averted, reductions in lifetime-risk for adolescent girls and young women, deaths averted, and Disability Adjusted Life Years (DALYs) averted. Adjustments will be made to model the costs as they would be in a ‘routine’ application, removing any elements that are exclusively required by the evaluation.” Study: *HIV prevention cascade* [20]
Effectiveness or efficacy	RE-AIM	“Success rate if implemented as in guidelines; defined as positive outcomes minus negative outcomes”	Individual patient	“We will test the following hypotheses: HIV−/u (HIV-negative or unknown) adolescent girls and young women from zones randomized to integrated wellness care + SHIELD will have higher HIV testing than adolescent girls and young women in zones randomized to SHIELD only or usual care. HIV+ (HIV positive) adolescent girls and young women from zones randomized to integrated wellness care + SHIELD will have higher retention in care and viral load suppression than adolescent girls and young women in zones randomized to SHIELD only or usual care.” Study: *Shield* [21]
Feasibility	Proctor	“…the extent to which a new treatment, or an innovation, can be successfully used or carried out within a given agency or setting (Karsh [70])”	Individual provider Organization or setting	“We will determine the primary outcomes of acceptability (quantitative assessment by Proctor/quantitative assessment of acceptability based on the UTAUT developed by Dr. Holden) [17, 22] and feasibility (enrollment, participation and completion thresholds > 80% for each).” Study: *InTSHA* [23]
Fidelity	Proctor	“…the degree to which an intervention was implemented as it was prescribed in the original protocol or as it was intended by the program developers (Dusenbury et al. [69]; Rabin et al. [68]).”	Individual provider	“Intervention fidelity is a key process evaluation indicator. For each group session, we will generate a list of session objectives that highlight the main knowledge, skills, and activities to be covered in each session. Using facilitator logs, facilitators will record whether the session objectives were met. When all objectives are not met, facilitators will be asked to explain why objectives were not covered in the session….Finally, the study coordinator will conduct random direct observations of sessions as another means of assessing fidelity and acceptability of intervention to participants using a semi-structured observation guide. These observations will also be used to provide feedback to the facilitators.” Study: *Family Connections *[24]
Implementation	RE-AIM	“Extent to which the intervention is implemented as intended in the real world”	Organization or setting	“Mentors in the intervention Safe Spaces will follow detailed guides for each adherence support club meeting during the study. Adherence to the guide will be monitored in person at each session by staff observers and documented on a standardized fidelity monitoring checklist. The fidelity monitoring checklist will also evaluate the quality of facilitation (e.g., facilitator maintains focus, nonjudgmental delivery), interaction with workshop participants to assess their engagement with the material, and any factors that may have affected implementation. The staff observer will also take field notes to document the discussion topics and questions that arose. Quality assurance and coaching meetings will be held after each session at each site to provide feedback and coaching on all intervention components to improve intervention delivery. Mentors will be retrained as necessary if the reviews indicate poor adherence to intervention protocols.” Study: *Tu’Washindi* [17]
Maintenance	RE-AIM	“Extent to which a program is sustained over time”	Individual consumer Organization or setting	“At the end of the primary intervention period and after the 6-month follow-up data has been collected, the primary intervention group will enter a maintenance phase and the comparison group will receive the intervention. This maintenance phase will consist of individual meetings and group meetings every other month. Youth at Arthur Davison Children’s Hospital who at this point are assessed as ready to transition physically to the adult care clinic will do so and have their maintenance phase at Ndola Teaching Hospital the adult clinic.” Study: *Project Yes!* [22]
Penetration	Proctor	“…the integration of a practice within a service setting and its subsystems.”	Organization or setting	“Implementation outcomes include intervention acceptability, feasibility, appropriateness, penetration, coverage, intervention fidelity, programme costs and cost-effectiveness (outcome definitions and measurement approach in Table 2). Healthcare workers will complete surveys during clinic continuous quality improvement meetings to determine the association between intervention adaptations and implementation outcomes. These surveys use validated measures of perceived appropriateness, acceptability, feasibility and fidelity of the adolescent transition package adaptations.” Study: *ATTACH* [25]
Reach	RE-AIM	“Proportion of the target population that participated in the intervention”	Individual patient	“A total of 58 youth received the Sauti ya Vijana intervention. The average age was 17.4 years; 15 (25.9%) were double orphans (both parents deceased), and 13 (22.4%) identified a grandparent as the primary caregiver (Table 2). The majority, 50 (86.2%), were perinatally HIV-infected. Approximately one-quarter reported having ever had sexual intercourse of whom 38.5% had disclosed their status to their partner.” Study: *Sauti ya Vijana* [25, 27, 28]
Sustainability	Proctor	“…the extent to which a newly implemented treatment is maintained or institutionalized within a service setting’s ongoing, stable operations.”	Administrators Organization or setting	“Sustainability will be evaluated at the end of the study by whether or not the host organization agrees to integrate the Peer Navigators program for the target population into their routine programming.” Study: *Peer Navigators* [29]

*Definitions are pulled directly from the referenced papers by Proctor et al. [14] or Glasgow et al. [15]

The coding team included four co-authors of this manuscript (KBS, SD, TC, IN), and two acknowledged researchers (SV, RS), who each participated in independent coding and code review. Each study document was independently coded by one author, and coded documents were reviewed by a second author. Disagreements were discussed and resolved through group discussion. Data were summarized using queries and code co-occurrence tables and re-presented in summary tables. Initially drafted summary tables were reviewed by three manuscript authors (IN, ADW, KBS) to ensure internal consistency in categorization across studies. Extracted and summarized data were returned to individual AHISA teams for review and verification of accuracy and completeness. In cases where the terminology between the study protocols and the review team’s conceptualization differed (e.g., defining an EBI versus strategy), the review team maintained its classification for internal consistency.

### Ethics

This study did not involve human subject data and was exempt from IRB research oversight.

## Results

This review focused on implementation outcomes, frameworks, and strategies applied to AYA HIV prevention and care among AHISA-affiliated studies. All 26 AHISA member study teams submitted one or more study protocols or protocol manuscripts, representing a total of 36 research studies. Studies represented ten countries; South Africa (10 studies [28%]), Kenya (6 [17%]), Zambia (3 [8%]), Tanzania (3 [8%]), Zimbabwe (3 [8%]), Nigeria (3 [8%]), and Uganda, Ghana, Botswana and Malawi (1 [3%] each). Four studies (11%) took place in multiple locations (Kenya and Canada, South Africa and Kenya, Malawi and South Africa, Kenya and Uganda). Fifteen studies (42%) focused on HIV prevention, 12 (33%) on HIV treatment, 3 (8%) on HIV testing, 3 (8%) studies on HIV treatment/testing/prevention, 2 studies on treatment/testing (6%), and 1 study (3%) on treatment/prevention. Of the 12 studies focused on HIV treatment, 4 were on transition to adult care, 2 on adherence alone, 3 on adherence and retention, 2 on mental health, and 1 on morbidity. Supplementary Table 1 summarizes strategies and outcomes across the HIV continuum of care.

### Study Designs

Randomized designs were most common, with 12 (33%) cluster randomized clinical trials (RCTs), 9 (25%) individual RCTs, 4 (11%) stepped wedge RCTs, 1 (3%) 2 × 2 factorial design RCT, 1 (3%) that included both stepped wedge and individually randomized RCT designs, 1 (3%) described as cluster RCT with individual randomization within clusters. There were 6 (17%) cohort studies, 1 (3%) described as observational, and 1 (3%) exclusively qualitative research design. Overall, 8 (22%) were defined as pilot studies.

Aligned with the broad research emphasis of AHISA, studies focused on a range of AYA populations, with 13 including AGYW, 17 including youth, 12 including YLH, either alone or in combination with caregivers and health providers, and 1 each including antenatal mothers, HIV negative male youth and health care workers (Table 2). YLH-defined populations spanned a range of age groups; the most common (12 [33]%) age groups were 14–25 years, while 8 studies (22%) included only youth ≤ 19 years of age.

**Table 2:** Study Descriptions of AHISA-affiliated studies

Short title (n = 36)	Population description	Population category	Geographic setting	Study design description	Study design category	EBP delivered	EBP category
3Ps for prevention (manuscript-2020) [41]	HIV-negative women (16–25)	AGYW	Cape Town, South Africa	Randomized evaluation of a behavioral economics intervention	Individual RCT	PrEP	Medication
3Ps for prevention (manuscript-2020) [35]	Young women age 16–25	AGYW	Cape Town, South Africa	PrEP demand creation campaign	Qualitative, pilot	PrEP	Medication
ACT Nigeria [66]	ALH ages 13–17 on ART	ALH	Six geopolitical zones across Nigeria	Hybrid type 1 effectiveness-implementation cluster randomized controlled trial	Cluster RCT	Structured transition program (Adolescent Coordinated Transition) with case management and peer support	Health system toolkit; Behavioral or social intervention
ATTACH (protocol manuscript-2020) [25]	HIV care clinics and adolescents (10–24) living with HIV	YLH, health providers	Homa Bay, Nairobi, Kajiado and Nakuru counties, Kenya	Hybrid type 1 effectiveness implementation cluster randomized controlled trial	Cluster RCT	Structured transition program, Disclosure booklet	Health systems toolkit
CHAMPS-MC- in (study protocol, 2013) [72]	HIV negative, adolescent males ages 14–17, parents/legal guardians	Other	Cape Town and Soweto, South Africa	Multi-site, mixed methods longitudinal cohort study	Prospective cohort	Male circumcision	Clinical services
CHAMPS-PrEP [61]	HIV-negative, sexually active youth ages 15–19	Youth	Cape Town and Johannesburg, South Africa	Open-label, phase 2 clinical trial	Observational study	PrEP, comprehensive prevention package	Medication
CHAMPS-Uchoose [62]	Sexually active, HIV negative female adolescents ages 15–19	AGYW	Cape Town, South Africa	Open-label randomized cross-over clinical trial	Individual RCT	Hypothetical PrEP (using contraception as a proxy)	Medication
Family connections (study protocol-2016) [24]	Adolescent/caregiver pairs	YLH; caregivers	Ndola, Zambia	Randomized pilot study	Individual RCT; pilot	Family-focused caregiver and ALHIV HIV support using an adapted WHO Positive Connections guide and corresponding newly developed caregiver guide	Behavioral or social intervention
Girls [63]	AGYW ages 15–24 without HIV at last test	AGYW	Western Kenya	Sequential, multiple assignment, pilot randomized clinical trial	Individual RCT, pilot	Multicomponent seek, test and link intervention that includes: (1) alternative recruitment strategies, (2) HIV testing options, (3) mobile reminders, and (4) financial incentives	Behavioral or social intervention; economic support
HIV prevention cascade: VMMC (2020 published protocol) [40]	HIV negative males age 15–29	HIV negative young males who are not circumcised	Manicaland province, Zimbabwe	Matched cluster RCT	Cluster RCT and individual RCT within cluster	VMMC	Clinical services
HIV prevention cascade: PrEP uptake (2019 published protocol) [20]	AGYW ages 18–24	AGYW	Manicaland province, Zimbabwe	Matched cluster RCT	Cluster RCT	PrEP	Medication
HIV testing [64]	Young women ages 18–26	AGYW	Mpumalanga Province, South Africa	1:1 individually randomized controlled trial	Individual RCT	HIV self-testing	Clinical services
InTSHA (study protocol, 2021) [23]	Perinatally HIV-infected adolescents (15–19) on ART and their primary caregivers, adolescent healthcare providers	YLH; caregivers, health providers	KwaZulu-Natal, South Africa	Pilot randomized controlled trial	Individual RCT; pilot	Behavioral intervention supporting transition to adult care, exploratory aim: stigma reduction intervention with motivational interviewing foundation but outcomes for this not tested	Behavioral or social intervention
MUHAS (COVID supplement) (Supplement protocol)	Out-of-school AGYW (15–24)	AGYW	Dar es Salaam, Tanzania	Mixed methods pilot study	Prospective cohort study	Social support networks (focused on social norm change and livelihood training)	Behavioral or social intervention & economic support
Ntemoga (published protocol 2020) [39]	Children living with HIV, HIV-exposed and uninfected children, HIV-unexposed and uninfected children (7–18)	YLH; CLH	Gaborone, Botswana	Prospective, observational cohort study	Prospective cohort study; pilot	Neurocognitive screening tool	Clinical services
Peer navigators (study protocol, 2020)	Street-involved and homeless youth (SIY) who identify as LGBTQ2S (16–29)	Youth	Toronto and Montreal, Canada Kitale and Eldoret, Kenya	Mixed methods evaluative longitudinal prospective study	Prospective cohort study	Comprehensive HIV care services	Medication; clinical services
POWER PrEP (study protocol-2016) [38]	HIV-negative women (16–25)	AGYW	Kisumu, Kenya; Johannesburg and Cape Town, South Africa	Prospective, observational, open-label cohort study	Prospective cohort study	PrEP	Medication
PrEP adherence + larger PrEP (Study protocol-2020)	Adolescent girls and young women ages 18–24 who are taking PrEP	AGYW	Homabay and Kisumu Counties, Kenya	Prospective mixed-methods study	Prospective cohort study	PrEP	Medication
PRISM Ghana [65]	Health providers from RCT facilities; cisgender HIV-negative or unknown HIV status MSM ≥ 18 years of age	Health providers, other	Ashanti and Greater Accra, Ghana	Wait-listed cluster randomized controlled trial among 4 size-matched healthcare facilities and individually randomized MSM	Cluster RCT	Multi-level, multi-component stigma reduction intervention	Behavioral or social intervention
PRIYA-SP (published protocol-2020) [43]	Healthcare workers and facilities where AGYW seek PrEP	Health providers	Nairobi, Kiambu, Kisumu, and Homa Bay, Kenya	Cluster randomized controlled trial	Cluster RCT	PrEP	Medication
Project YES! (study protocol 2018) [22]	Youth living with HIV (15–24) on cART	YLH	Ndola, Zambia	Individual-level randomized controlled trial with a stepped wedge design	Stepped wedge RCT	AIDSTAR-One toolkit for supporting youth transitioning to independent care as well as their caregivers	Health systems toolkit
SA IMARA (study protocol 2019) [19]	Black and mixed race (Xhosa speaking) South African AGYW (15–19) and their female caregiver-dyads	AGYW; caregivers	Cape Town, South Africa	2-arm individually randomized controlled trial	Individual RCT	Adapted IMARA: strengthen AGYW-caregiver relationships/communication about STI/HIV prevention and safer sexual behavior; increase self-efficacy to use condoms; improve caregiver monitoring of AGYW activities; distinguish healthy and unhealthy relationships; improve emotion regulation; promote pride in South African female culture; encourage gender empowerment	Behavioral or social intervention
Sauti ya Vijana (study protocol 2015 AND 2 manuscripts with pilot results) [26–28]	Youth living with HIV (12–24) on ART	YLH	Moshi, Tanzania	Individual-level randomized pilot with stepped wedge design (2020) and individual randomized trial (2019)	Stepped wedge RCT, individually randomized RCT, pilot	Multi-component mental health intervention: components of trauma informed CBT, interpersonal psychotherapy, and motivational interviewing	Behavioral or social intervention
Sauti ya Vijana Scale (study protocol 2021) [28]	Youth living with HIV (10–24) on ART	YLH	Kilimanjaro, Mwanza, Mbeya, Ifakara, Tanzania	Individual-level randomized parallel arm RCT	Individual randomized RCT	Multi-component mental health intervention: components of trauma informed CBT, interpersonal psychotherapy, and motivational interviewing	Behavioral or social intervention
SEARCH Youth (Study protocol, 2019)	YLH ages 15–24 attending HIV care; health providers from RCT facilities, family members of participating youth	YLH, Health providers, caregivers	Western Kenya; Southwestern Uganda	Clinic-based cluster randomized clinical trial of 28 clinics; balanced by country and clinic size	Cluster RCT	SEARCH Youth (combination, multi-component intervention including (1) life stage assessment, (2) identification of clinic access choices, (3) rapid viral load results, and (4) provider whatsapp collaboratives)	Health Systems Toolkit; Behavioral or social intervention
SHIELD (published protocol 2019) [21, 67]	AGYW (16–24) diagnosed with HIV in past 3 years, AGYW (10–20) who are HIV negative	AGYW	Lusaka, Zambia	Clinic-level randomized controlled trial	Cluster RCT	Multi-component EBPs: SHIELD intervention (support for HIV integrated education, linkages to care and destigmatization), integrated wellness care clinic,	Behavioral or social intervention
SMM for HIV testing ((Study protocol, 2015)	Youth 16–24 attending clinic with cell phones or social media	Youth	Chitungwiza, Zimbabwe	Interrupted time series with a community comparison	Cluster RCT	Social media/marketing	Behavioral or social intervention
SPEED (published protocol 2017) [44]	HIV care clinics and adolescents (10–24) living with HIV	YLH; health providers	Nairobi, Kiambu, Kisumu and Homa Bay counties, Kenya	Stepped-wedge RCT	Stepped wedge RCT	ART	Medication
Suubi adherence (published protocol 2019) [58]	Youth living with HIV (10–16) enrolled in ART care	YLH	Greater Masaka Region of Uganda	2-arm cluster randomized-controlled trial	Cluster RCT	Multi-component family based economic empowerment: Workshops on asset building, mentorship, child development accounts, family income generating activity promotion	Economic support
Thetha Nami (study protocol 2020) [18]	Young people (16–29)	Youth	KwaZulu-Natal, South Africa	Mixed method process evaluation	2 × 2 factorial design RCT with a process evaluation	Multi-component Holistic EBPs: Universal test and treat (UTT), PrEP, and FP	Medication; clinical services
Tsima community mobilization [71]	Community residents ages 18–49 years of age	Other	Mpumalanga, South Africa	Community-based cluster randomized clinical trial	Cluster RCT	Community mobilization	Behavioral or social intervention
Tu’Washindi (study protocol 2019) [17]	AGYW ages 15–24 enrolled in the DREAMS Initiative	AGYW	Siaya County, Kenya	Mixed methods; pilot using cluster-randomized controlled trial design	Pilot; cluster RCT	PrEP	Medication
4YBY (published protocol 2021) [48]	Youth age 14–24 who have a mobile phone	Youth	Nigeria	Type 1 hybrid effectiveness-implementation trial	Stepped wedge cluster RCT	HIV self testing	Clinical service
iCARE (study protocol 2021) [46]	Youth age 15–24 living with HIV, Male youth age 15–24 not living with HIV	YLH; Youth	Nigeria	Stepped wedge cluster RCT	Stepped wedge cluster RCT	HIV testing and treatment	Medication; clinical services
Option B + (study protocol 2019) [59]	Couples age 15+	Antenatal mothers and partners	Malawi	RCT comparing a couple-based intervention to individual based standard of care	Individually randomized RCT	PMTCT	Medication
Girl Power (published protocol 2017) [60]	AGYW age 15–24	AGYW	Malawi and South Africa	Pilot cluster RCT	Cluster RCT, pilot	Comprehensive SRH	Clinical services

### Evidence-Based Interventions

There was diversity in the types of EBIs delivered in the 25 studies. Broadly, these were classified into 14 (39%) studies delivering medications (PrEP and ART), 13 (36%) delivering behavioral or social interventions, 9 (25%) delivering clinical services beyond medication, 4 (11%) delivering health systems toolkits, and 2 (5%) providing economic support. A few studies used a combination of EBIs as their intervention. These studies combined EBIs across categories, including 3 studies that combined medication and clinical services and 1 study that combined behavioral or social interventions with economic support. Other studies evaluating combined EBIs integrated multiple EBIs from the same category (e.g., behavioral/social EBIs) into a single multicomponent EBI approach for the study.

These multicomponent EBI approaches are useful for strengthening the effect of a therapy on a single health outcome or to broaden the number of health outcomes targeted in the EBI package. For example, in the Sauti ya Vijana pilot [26, 27] and scale up study [28], a multicomponent behavioral/social EBI included components of trauma-informed cognitive behavioral therapy, interpersonal psychotherapy, and motivational interviewing, all unique mental health therapy EBIs focused on achieving specific mental health outcomes. In another multi-component approach, the Thetha Nami study [18] delivered a multicomponent clinical services and medication EBI, including universal test and treat, family planning, and pre-exposure prophylaxis, to reach a broader range of HIV and sexual and reproductive health outcomes.

### Implementation Outcomes, Determinants, and Frameworks

**Fig. 1:**
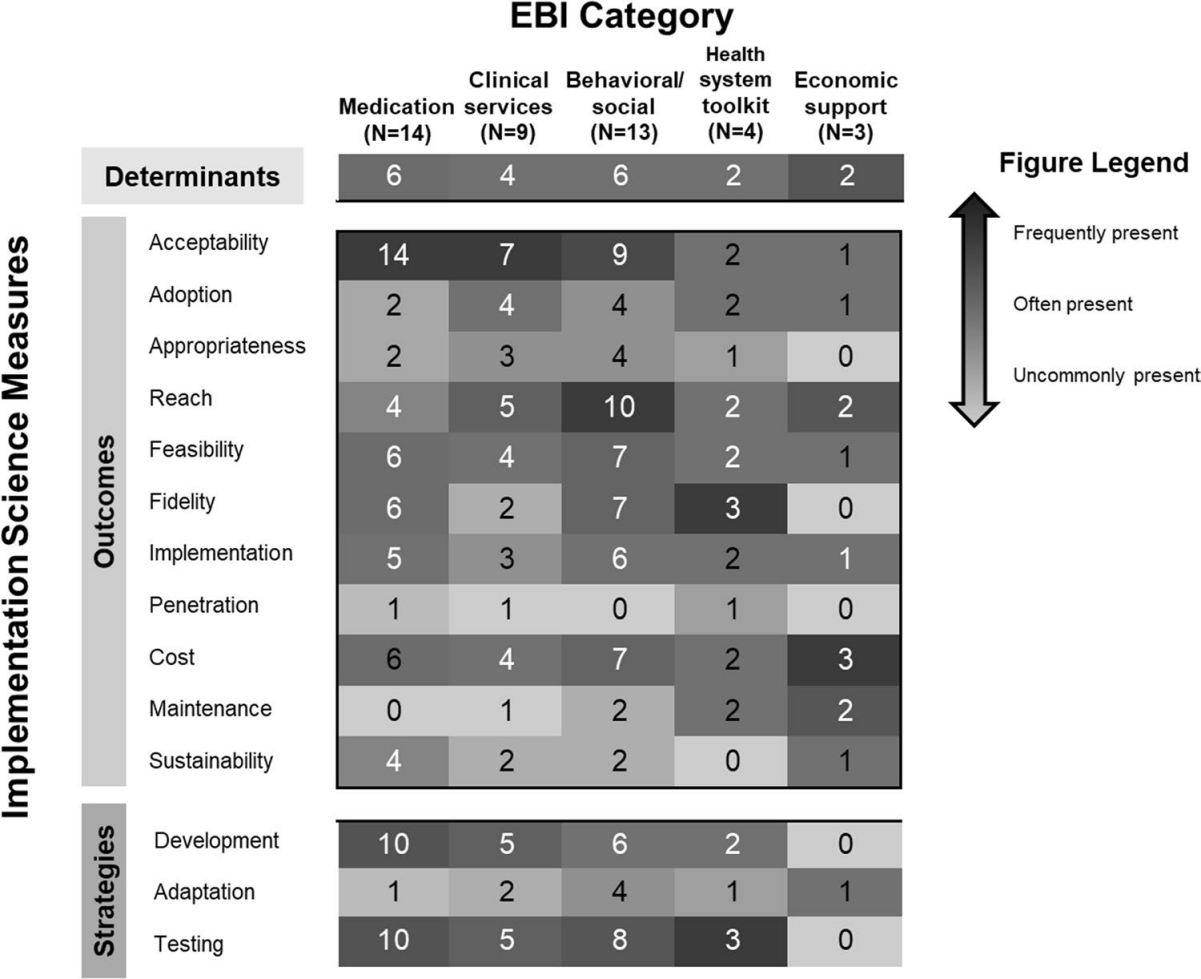
Characterization of coverage in measurement of IS outcomes and strategies by EBI. AHISA study EBIs were classified into five representative categories. IS outcomes were listed by stage of implementation, ranging from early (acceptability, adoption, appropriateness, reach, feasibility), to mid (fidelity, implementation, penetration, cost), and late (maintenance, sustainability) stages. Presence or absence of specific implementation outcomes and strategies was assessed within each EBI category and organized into a heat map representing the overall evidence available for each implementation measure

Implementation outcomes were defined by Proctor’s IOF and RE-AIM. The definitions and example quotes for how each outcome was operationalized within study protocols are summarized in Table 1. All 36 studies measured at least one implementation outcome. The most commonly measured outcomes were acceptability (n = 29), implementation (n = 13), feasibility (n = 16), cost (n = 16), fidelity (n = 15), and reach (n = 17) (Table 3). Outcomes measured less commonly included appropriateness (n = 8), adoption (n = 9), sustainability (n = 6), maintenance (n = 5), and penetration [2] (Table 3). Earlier phase implementation outcomes (e.g., acceptability, feasibility, appropriateness, adoption) were more common across the studies than later phase outcomes (e.g. sustainability, maintenance) (Fig. 1). The operationalization of these outcomes was heterogeneous, and there were few occurrences in which a validated implementation outcome measure was utilized or utilized consistently across studies. Studies that focused on the same aspects of the HIV care continuum assessed IS outcomes at different timepoints, among different stakeholder groups and using different measurement tools. For example, the InTSHA and ATTACH studies both focused on transition to adult care and measured acceptability. However, the InTSHA study measured acceptability among those receiving the intervention using the Unified Theory of Acceptance and Use of Technology (UTAUT) [31, 32], while the ATTACH study measured acceptability among those delivering the intervention using the Acceptability of Intervention Measure [34].

**Table 3:** Study Implementation Characteristics of AHISA-affiliated studies

Short title (n = 36)	Implementation outcomes	Clinical outcomes	Implementation strategies tested	Strategy category	Determinants/barriers and facilitators studied	IS frameworks or models used
3Ps for prevention (manuscript-2020) [41]	Acceptability, implementation	PrEP use, PrEP adherence, HIV status	Conditional financial incentive (drug level feedback offered to both intervention and control arms)	Develop, test	None	None
3Ps for prevention (manuscript-2020) [35]	Acceptability	None (precursor outcomes—PrEP interest and knowledge—were tested)	Demand creation: PrEP video and brochures	Develop, test	Identify determinants of PrEP for AGYW	Behavioral centered design framework for designing behavior change interventions
ACT Nigeria [66]	Adoption, effectiveness/efficacy, implementation, maintenance, reach	Post-transition retention at 12 and 24 months; viral suppression, psychosocial wellbeing	None	N/A	None	RE-AIM
ATTACH (protocol manuscript-2020) [25]	Acceptability, appropriateness, cost, coverage, feasibility, fidelity, penetration, reach	Transition readiness (precursor outcome), retention, viral suppression	Booklets and tracking tools: these were developed and adapted for the setting. The disclosure intervention was adapted	Develop, adapt, test	Identify determinants of implementation	CFIR, FRAME
CHAMPS-MC- in (study protocol, 2013) [72]	Acceptability	Uptake of male circumcision; correlates of male circumcision	None	N/A	None	None
CHAMPS-PrEP [61]	Acceptability	PrEP uptake; PrEP Adherence; PrEP Persistence	None	N/A	None	None
CHAMPS-Uchoose [62]	Acceptability	HIV prevention method preference; method adherence; HIV status, pregnancy, STIs	None	N/A	None	None
Family connections (study protocol-2016) [24]	Acceptability, adoption, appropriateness, feasibility, fidelity, implementation, reach	ART adherence, viral load	Support groups for adolescents and caregivers	Develop, adapt, test	Identify how determinants of stigma (an exposure of interest) operate at individual vs group level	(Not IS specific model): analysis, design, develop, implement, evaluate (ADDIE) model to develop the manuals
Girls [63]	Cost, effectiveness/efficacy, reach	Preferred testing recruitment venue; preferred HIV testing strategy; HIV status, linkage to care; Retention in care; Linkage to prevention services	Home-based and community-based recruitment	N/A	None	None
HIV prevention cascade: VMMC (2020 published protocol) [40]	Acceptability, cost, effectiveness or efficacy, feasibility, reach	VMMC uptake, HIV risk perception, self reported sexual behavior change	Peer led education sessions, incentives, community engagement through community conversations	Develop, test	Identify determinants of prevention EBIs	(Not IS specific model): HIV prevention cascade framework was used to guide identification of relevant responses and platforms for interventions
HIV prevention cascade: PrEP uptake (2019 published protocol) [20]	Acceptability, cost, effectiveness or efficacy, feasibility, reach	PrEP uptake, risk perception, self reported change in sexual behavior	PrEP demand creation: interactive digital tablet quiz on HIV risk and active counseling on PrEP, community engagement through community conversations	Develop, test	Identify determinants of PrEP uptake, specifically social and cultural barriers	(not IS specific model): HIV prevention cascade framework was used to guide identification of relevant responses and platforms for interventions
HIV testing [64]	Adoption, effectiveness or efficacy	HIV test use; test distribution to peers/partners; HIV status	None	N/A	None	None
InTSHA (study protocol, 2021) [23]	Acceptability, effectiveness or efficacy, feasibility, implementation, reach	Viral load, retention, precursor outcomes (peer connection, connection to clinical staff)	Social media delivery	Develop, test	Determine how implementation strategy (social media) addresses determinants to transition care	Proctor—they also use the socio-ecological model of adolescent and youth readiness to transition to develop the intervention
MUHAS (COVID supplement) (Supplement protocol)	Acceptability, adoption, cost, effectiveness or efficacy, feasibility, implementation, maintenance, reach, sustainability	None (precursor outcomes—HIV risk and vulnerability—measured)	No strategy, but did adaptations for COVID-19 (masking, hand sanitizer, social distancing)	Adapt	Identify determinants	RE-AIM
Ntemoga (published protocol 2020) [39]	Acceptability, appropriateness	Morbidity	No strategy tested—adaptation of the neurocognitive screening tool	Adapt	None	None
Peer navigators (study protocol, 2020)	Acceptability, adoption, appropriateness, cost, effectiveness or efficacy, feasibility, implementation, penetration, reach, sustainability	Medication adherence, viral load	No strategy tested but a peer support strategy was adapted	Adapt	Identify determinants of HIV prevention, testing and treatment	None
POWER PrEP (study protocol-2016) [38]	Acceptability, cost, effectiveness or efficacy, feasibility, implementation, sustainability	Medication adherence, PrEP use, morbidity, viral load	Training on PrEP delivery and technical assistance for integrating PrEP delivery to other services	Develop, test	Identify determinants of PrEP adherence	None
PrEP adherence + larger PrEP (Study protocol-2020)	Acceptability	PrEP Adherence, PrEP program persistence	None	N/A	None	None
PRISM Ghana [65]	Acceptability, effectiveness/efficacy, feasibility	HIV status; intersectional stigma	None	N/A	None	ADAPT-ITT
PRIYA-SP (published protocol-2020) [43]	Acceptability, fidelity	Service outcomes—patient-centeredness (interpersonal skills, communication skills) Effectiveness (overall quality—including both communication skills and adherence to guidelines)	Simulated patient training	Develop, test	None	Proctor
Project YES! (study protocol 2018) [22]	Acceptability, adoption, feasibility, fidelity, implementation, maintenance	Viral load, adherence	Task shifting and supervision: Peer mentoring strategy with 2 week training for peer mentors to deliver the intervention and continued supervision and practice	Develop, test	No determinants investigated (additional investigation to identify mechanisms of action to enhance adolescents’ viral suppression)	None
SA IMARA (study protocol 2019) [19]	Acceptability, adoption, appropriateness, cost, effectiveness or efficacy, fidelity, implementation, reach, sustainability	HTC/PrEP/STI uptake; STI incidence, HIV incidence, non condom use, number of partners	Task shifting, training and supervision: there was adaptation of the intervention and detailed manual and facilitator guide as well as weekly supervision to deliver the intervention	Adapt, test	No determinants investigated (additional investigation to identify moderators and mediators of action)	EPIS
Sauti ya Vijana (study protocol 2015 AND 2 manuscripts with pilot results) [26–28]	Acceptability, appropriateness, effectiveness or efficacy, feasibility, fidelity, implementation, reach, cost	Mental health, Medication adherence, viral load, emotional and behavioral functioning, sexual risk and drug use	Task shifting and training: lay counselors/peer leaders trained in CBT and intervention delivery	Develop, test	Identify determinants of successful introduction of the implementation strategy (peer educators)	Intervention is based on the social action theory
Sauti ya Vijana Scale (study protocol 2021) [28]	Acceptability, cost, effectiveness or efficacy, feasibility, fidelity, reach, adaptations	Viral suppression, mental health, coping, resilience, stigma, quality of life, gender based violence, disclosure, HIV knowledge, high risk behaviors	Training and supervision: there was an elaborate training system and weekly supervision. The intervention was delivered by peers living with HIV (age 23–29)	Develop, test	Identify determinants of implementation of EBI and implementation strategy	CFIR (updated protocol), FRAME for adaptations
SEARCH Youth (Study protocol, 2019)	Cost, effectiveness/efficacy, fidelity	Viral suppression; retention	Community-based HIV testing; provider training and feedback	Test	Identify determinants of fidelity to EBP	PRECEDE
SHIELD (published protocol 2019) [21, 67]	Acceptability, appropriateness, cost, effectiveness or efficacy, fidelity	HIV testing, retention in care, Medication adherence, viral load, HIV risk behaviors. Intermediate outcomes: self efficacy, social support, mental health, HIV stigma, gender based violence, unintended pregnancy	Task shifting and training: there were adaptations to existing EBPs to comprise the final package of the intervention. Some of the intervention was peer delivered through youth clubs facilitated by peer navigators	Develop, adapt, test	None	Proctor
SMM for HIV testing ((Study protocol, 2015)	Acceptability, effectiveness or efficacy, feasibility, reach	HIV status; new patients seeking testing, clinic visitation by high-risk youth	Peer driven HIV testing campaign using word of mouth (WOM); peers and SMS messages grounded in social cognitive theory	Develop, test	None	None
SPEED (published protocol 2017) [44]	Acceptability, cost, effectiveness or efficacy, fidelity, sustainability	Retention, adherence, viral suppression; service outcomes: patient-centeredness (interpersonal skills, communication skills)	Simulated patient encounters	Develop, test	None	Proctor
Suubi adherence (published protocol 2019) [58]	Cost, effectiveness or efficacy, maintenance	Medication adherence, retention, viral load, intermediate outcomes (family financial stability, mental health functioning, sexual risk behaviors, HIV knowledge)	No strategy tested	N/A	Identified determinants of adherence to HIV treatment	None
Thetha Nami (study protocol 2020) [18]	Acceptability, appropriateness, cost, effectiveness or efficacy, feasibility, fidelity, implementation, reach, sustainability	Linkage to care, medication adherence, morbidity, viral load, new HIV diagnosis, uptake of PrEP and universal test and treat and population viral load, SRH outcomes: new pregnancy, STIs, mental health outcomes, retention, adverse events	Multi-component implementation strategy: Peer navigator intervention, adolescent and youth friendly clinics, home based STI self sampling	Develop, test	None	Behavior change wheel, acceptability, practicability, effectiveness, affordability, safety/side effects, equity (APEASE) criteria (looks like a process evaluation framework used to evaluate appropriateness of an intervention)
Tsima community mobilization [71]	Effectiveness or efficacy, fidelity, reach	HIV status; linkage to care; retention	None	N/A	None	None
Tu’Washindi (study protocol 2019) [17]	Acceptability, effectiveness or efficacy, feasibility, fidelity, implementation	PrEP uptake, PrEP adherence, PrEP persistence, safety of intervention delivery	Multi-component strategy to improve PrEP uptake among AGYW at risk of HIV: community level PrEP sensitization for men, buddy day event for couples and PrEP support clubs	Develop, test	Identify determinants of EBI (PrEP) use	None
4YBY (published protocol 2021) [48]	Adoption, effectiveness or efficacy, implementation, maintenance, reach, economic evaluation	Uptake of HIVST, facility based HIV testing, STI testing and treatment, linkage to youth friendly services, PrEP referral, condomless sex, youth engagement	Multi-component strategy to increase HIVST uptake: (1) HIVST bundle containing HIVST kits and photo verification system, (2) participatory learning community, (3) peer to peer support and technical assistance, (4) On site supervision and performance feedback	Develop, test	Identify determinants of EBI (HIVST) uptake	RE-AIM
iCARE (study protocol 2021) [46]	Acceptability, adoption, cost,, effectiveness or efficacy, feasibility, fidelity, reach, intervention readiness	HIV testing: HIV prevalence, linkage to care among young men HIV treatment: viral suppression, medication adherence and retention	Combination demand creation and service delivery strategies: social media engagement (SMS—daily, free, bidirectional, personalized based on CDC recommended TXTXT) delivered by peers and Peer Navigation (trained peer navigators)	Develop, test	Identify determinants of readiness for implementation strategy (iCARE)	RE-AIM, CFIR
Option B + (study protocol 2019) [59]	Acceptability, fidelity	Female viral suppression; HIV testing among male partners, ART initiation and retention among men, viral suppression among men, uptake of circumcision among men, unprotected sex, EID uptake	Couple-based approach, including: assisted partner notification, enhanced couple counseling and testing, co-location of ART services for both couple members	Develop, test	None	None
Girl Power (published protocol 2017) [60]	Acceptability (qualitative sub-study assessing youth acceptability with implementation strategies)	Care seeking behaviors—HTC, condoms, FP, STI services, HIV risk behaviors	Multi-component strategy: (1) Youth friendly services, including community outreach, peer navigation, provider training, integrated services, (2) manualized facilitator led small group interactive sessions on behavioral interventions, (3) conditional cash incentives conditional on attending the monthly empowerment sessions	Develop, test	None	None

All studies measured clinical outcomes or precursors to clinical outcomes alongside implementation outcomes, representing reliance on hybrid effectiveness-implementation trial designs. The clinical outcomes measured aligned closely with the EBIs being tested. Many studies included precursor outcomes that were proximal to clinical outcomes of interest. For example, the 3P study included PrEP interest and knowledge as precursors to PrEP uptake or adherence [35] and the ATTACH study measured transition readiness as a precursor to successful transition [25] (Table 3).

Less than half of studies (n = 16) assessed determinants of implementation of EBIs, and a few explored how specific strategies might overcome specific barriers. For example, the 3P study [35], the HIV prevention cascade study [20, 40], POWER PrEP [38] and Tu’Washindi [17] assessed barriers to PrEP at the individual, social, and cultural levels. The InTSHA study [23] focused on assessing how their social media implementation strategy overcame specific barriers and enhanced facilitators to transition care. While not specifically related to determinants, two studies described investigating mechanisms, mediators, and moderators (Project YES! [22] and SA IMARA [19] (Table 3)) of EBI implementation.

Only half (n = 19) of the studies specifically mentioned applying a framework, model, or theory to inform their studies. The most common were RE-AIM (n = 4) [15], the Consolidated Framework for Implementation Research (CFIR) (n = 3) [45], the FRAME (n = 2) [30] used to track adaptation, Proctor’s IOF (n = 4) [14], and the Exploration, Preparation, Implementation, Sustainment (EPIS) framework (n = 1), and PRECEDE (n = 1) [42]. Seven studies employed frameworks or theories that were not explicitly implementation science frameworks, including those focused on behavioral theories, like the HIV Prevention Cascade framework [36]. Of note, many studies utilized outcomes language from either RE-AIM or Proctor’s IOF without specifically mentioning these frameworks in their protocols (Table 3).

### Implementation Strategies

Across AHISA, 26 studies incorporated one or more implementation strategies and 21 studies developed and tested a strategy. For example, the 3P study developed and tested a conditional financial incentive based on PrEP drug levels to motivate adherence [41], while the iCARE study developed and tested a combination demand creation and service provision implementation strategy that included personalized interactive SMS support and peer navigation [46]. Seven studies engaged in adapting an EBI; 3 only adapted an EBI while 4 adapted and tested an EBI. Adaptation was more common among the behavioral and social EBIs. For example, the MUHAS study did not test a strategy but did describe adapting the EBI to be delivered while observing COVID-19 prevention measures [47]. In contrast, the ATTACH study engaged in adapting an EBI disclosure toolkit, developing a transition toolkit, and testing the combined package with a strategy of tracking and training tools [25]. Testing strategies was most common among studies delivering medication EBIs (Fig. 1). When mapped to ERIC, implementation strategies were predominantly targeting change at the interpersonal level, including provider changes in training (e.g., use of training manuals, tracking sheets, and patient actors for simulation-based training), task shifting (e.g., to peers or lay counselors), and supervision. For studies delivering PrEP, the strategies tested occurred at different levels, including incentives (individual level), video and brochure education (individual level), interactive counseling (interpersonal level), and mobilization and community engagement (community level).

## Discussion

This review of AHISA protocols and studies revealed a rich body of implementation science focusing on HIV prevention and HIV care interventions for AYA populations in high HIV-burden African countries. Most studies focused on early implementation outcomes of delivering medication, clinical, and behavioral/social EBIs and all used a hybrid trial approach that included measurement of clinical outcomes. The use of frameworks and assessment of determinants was reasonably common, but fewer studies utilized validated implementation outcome measures. Many studies delivered EBIs in parallel with an implementation strategy, with some experimentally testing strategies. Formal evaluation of mechanisms, moderators, and mediators of EBI implementation was uncommon.

Since the original formation of the AHISA in 2017, the use of frameworks, measurement of implementation outcomes, and testing of implementation strategies has expanded in NIH’s implementation science portfolio [12]. Facilitating this expansion, as part of the AHISA collaboration, study teams received intensive implementation science training to strengthen current research designs and inform future IS grants. Expanded training in IS among AHISA teams was reflected in the shared research protocols, with increasing use of IS frameworks in the most recently developed protocols. For example, the Sauti Ya Vijana scale protocol [28] included the CFIR framework to evaluate barriers and facilitators to implementation and the FRAME to evaluate intervention adaptations, expanding IS activities from those included in the earlier pilot [26, 27]. Additionally, almost all AHISA-related protocols dated 2020–2021 included a formal IS frameworks (CFIR, RE-AIM, FRAME, Proctor) [23, 28, 46, 48], whereas most protocols dated 2017–2019 did not. This extended use of IS frameworks among AHISA team research projects demonstrates progress towards achieving the AHISA goal of building implementation science capacity among adolescent HIV researchers in high HIV-burden African countries [12]. As implementation of HIV prevention and care interventions for AYA populations continues and moves from early- to mid- to late-implementation, we expect the AHISA portfolio to grow to include later stage implementation outcomes (e.g., sustainability and penetration) in addition to early implementation outcomes (e.g., acceptability and feasibility) that are common in the current portfolio. Similarly, we expect more studies to shift beyond identifying barriers to implementation and instead focus on testing implementation strategies. A series of similarly structured reviews of interventions addressing stigma [49], non-communicable diseases [50], and depression [51] in resource-limited settings observed few studies that measured later implementation outcomes, and had less specification and testing of implementation strategies, and suboptimal usage of implementation frameworks.

In this review, many studies included an implementation strategy, but often the strategy was not referred to using IS strategy terminology in the protocol. This represents an opportunity to strengthen future research in this area; operationalizing strategies using Proctor’s specification scheme [52] will contribute to the growing evidence linking specific IS strategies to particular outcomes. Additionally, many studies that utilized a strategy did not test the impact of the strategy on implementation outcomes experimentally (a traditional implementation study) but rather conducted hybrid effectiveness-implementation type I designs with clinical outcomes as the primary focus and inclusion of implementation outcomes [53]. As time progresses, we expect more research to employ hybrid type II (equal focus on clinical and implementation outcomes) and III designs (primary focus on implementation outcomes with inclusion of clinical outcomes), as well as purely implementation foci. Finally, most of the implementation strategies tested focused on interpersonal level changes, with the exception of studies focused on PrEP delivery, which included strategies at individual, interpersonal, and community levels. One gap that could be strategically addressed in future HIV prevention research would be testing implementation strategies at higher levels for non-PrEP EBIs. These could include systems-level and community-level strategies, which are well suited to achieve later implementation outcomes like sustainability and penetration. In a similar review of implementation science applied to PrEP delivery for pregnant and postpartum populations, the authors focused on earlier implementation outcomes. They noted fewer studies testing implementation strategies, and of those strategies being tested, fewer tested systems-level or higher level strategies [54].

Adaptation of EBIs was common in the AHISA-affiliated studies. Many interventions required adaptation to a different cadre of provider (often shifting to peers), a new population (e.g., AYA instead of adults) or context (shifting from in-person to mobile delivery), and often to settings with fewer resources than the ones where the EBI was originally developed and tested. Despite adaptation being common, only two studies (Sauti ya Vijana [28] and ATTACH [25]) utilized a published framework to structure the documentation of the adaptation process (the FRAME framework [30]). Most AHISA studies were affected by the COVID-19 pandemic during study implementation, which presented an opportunity to adapt intervention delivery rapidly and creatively to new platforms, such as mobile delivery of the ATTACH and MUHAS interventions [25, 47]. Given the dynamic nature of intervention implementation over time [55] and the need to be responsive to unanticipated circumstances, systematic evaluation of adaptations are critical to understand intervention optimization within given contexts as AYA research places greater focus on sustainability and scale-up.

Within implementation science, timely methodologic challenges include development and psychometric validation of implementation measures for contexts outside the US and Canada [10, 11, 56], as well as elucidating implementation strategy mechanisms and identifying moderators and mediators that activate or inhibit mechanisms [57]. Future implementation science projects in resource-limited settings have an opportunity to advance these scientific and pragmatic areas. Two studies in this review included mechanism, moderator, and mediator language. Similarly, few studies utilized validated implementation outcome measures like the acceptability, appropriateness, and feasibility measures by Weiner et al. [34]. This limited use may be warranted given the dearth of context-validated measures at this point in time. For example, one study that formally adapted and assessed validity of an implementation determinant measure of organizational readiness found that several new domains were required to reflect structural context [33], while a review and application of the CFIR to LMICs revealed the need to add a new domain and new constructs to improve compatibility for use in LMICs [37].

This review is limited in several ways. We only included studies affiliated with AHISA study teams. We did not undertake either a systematic review of all AYA HIV IS research nor a structured review of all NIH-funded studies in this area. The findings of this review are not generalizable to the broader arena of AYA HIV IS research. Some of the AHISA studies were designed when there was less discussion about the importance of harmonization, the application of implementation frameworks, the selection and operationalization of implementation outcomes, and the selection and testing of implementation strategies. As a result, much of the categorization of these items was completed by our team and may differ from how study teams might characterize their work. However, we provided study teams the opportunity to check all categorization in this manuscript to ensure accuracy. Additionally, it is a testament to the capacity-building impact of the AHISA program that protocols developed by teams after AHISA supported IS training incorporated many of these newer practices. Finally, due to less specification of implementation strategies within protocols, it was not possible to map strategies to an orienting list, such as the ERIC [16].

## Conclusion

Current AHISA supported research delivers diverse EBIs and measures a range of clinical and implementation outcomes. Future studies that address lack of measurement harmonization across studies and focus on developing and validating implementation measures in heterogeneous contexts could improve development of an implementation-related foundation and improve cross-study comparisons. Additional opportunities for advancing the agenda of AYA HIV IS research include expanding the selection, specification, and testing of implementation strategies beyond the individual and interpersonal, documenting the motivation and results of adaptation of EBIs to new populations and contexts, especially resource-constrained settings, and expanding the scope of inquiry to include identification of mechanisms of action.

## Supplementary Information

Below is the link to the electronic supplementary material.

Supplementary file1 (DOCX 78 KB)

## Data Availability

The majority of data is available through research article databases and clinicaltrials.gov. Non-publicly available protocols can be requested by contacting the lead author and PI of the AHISA-affiliated research project.
