# Supplementary material for: Addressing the Know-Do Gap in Adolescent HIV: Framing and Measuring Implementation Determinants, Outcomes, and Strategies in the AHISA Network
Source: AIDS Behav. 2023 Mar 11;27(Suppl 1):24–49. doi: 10.1007/s10461-023-04021-3 (PMC10007651; doi:10.1007/s10461-023-04021-3)
Supplement: Supplementary file 1 — Supplementary file1 (DOCX 78 KB) [file 10461_2023_4021_MOESM1_ESM.docx]

**Supplementary Table 1: Prevalence of Implementation Science Outcomes by HIV Care Continuum Categories among AHISA studies**

|  | HIV prevention  (n=15) | HIV treatment  (n=12) | HIV testing  (n=3) | Prevention testing and treatment  (n=3) | Treatment and testing  (n=2) | Prevention and treatment  (n=1) |
| --- | --- | --- | --- | --- | --- | --- |
|  | | | | | | |
| Acceptability | 14 | 10 | 2 | 2 | 0 | 1 |
| Adoption | 3 | 4 | 1 | 1 | 0 | 0 |
| Appropriateness | 0 | 6 | 0 | 2 | 0 | 0 |
| Cost | 5 | 7 | 0 | 3 | 1 | 0 |
| Feasibility | 6 | 6 | 2 | 2 | 0 | 0 |
| Fidelity | 3 | 8 | 0 | 1 | 2 | 1 |
| Implementation | 5 | 6 | 0 | 2 | 0 | 0 |
| Penetration | 0 | 1 | 0 | 1 | 0 | 0 |
| Maintenance | 2 | 3 | 0 | 0 | 0 | 0 |
| Reach | 5 | 7 | 1 | 3 | 1 | 0 |
| Sustainability | 2 | 2 | 0 | 2 | 0 | 0 |
|  | | | | | | |
| Develop | 10 | 8 | 1 | 1 | 0 | 1 |
| Adapt | 1 | 5 | 0 | 1 | 0 | 0 |
| Test | 11 | 9 | 1 | 1 | 1 | 1 |
